# Genipin, an Inhibitor of UCP2 as a Promising New Anticancer Agent: A Review of the Literature

**DOI:** 10.3390/ijms23105637

**Published:** 2022-05-18

**Authors:** Young Seok Cho

**Affiliations:** Department of Nuclear Medicine, Samsung Medical Center, Sungkyunkwan University School of Medicine, Seoul 06351, Korea; ysnm.cho@samsung.com; Tel.: +82-2-3410-6290; Fax: +82-2-3410-2638

**Keywords:** genipin, uncoupling protein-2, cancer cell metabolism, reactive oxygen species

## Abstract

Genipin is a protein cross-linking agent extracted from Gardenia (*Gardenia jasminoides* Ellis) fruits. This fruit has conventionally been used as a Chinese herbal medicine for the treatment of inflammation and jaundice and as an edible colorant in oriental countries. Uncoupling protein (UCP)-2 is a member of the family of uncoupling proteins, which are anion transporters positioned in the mitochondrial inner membrane. Genipin has been shown to have hepatoprotective activity, acting as an effective antioxidant and inhibitor of mitochondrial UCP2, and is also reported to exert significant anticancer effects. In this review, the author presents the latest progress of genipin as an anticancer agent and concisely describes its various mechanisms of action. In brief, genipin inhibits UCP2 to attenuate generation of reactive oxygen species (ROS), leading to ROS/c-Jun N-terminal kinase-dependent apoptosis of cancer cells. Genipin also increases the tissue inhibitors of matrix metalloproteases (MMP)-2, a kind of tumor promoter in a variety of cancers, as well as induces caspase-dependent apoptosis in in vitro and in vivo models. These findings suggest that genipin can serve as a promising novel antitumor agent that could be applicable for chemotherapy and/or chemoprevention for cancers.

## 1. Introduction

Cancer cells exhibit a multitude of mitotic, biochemical, and cellular aberrancies. Many solid tumors have been shown to exhibit an increased dependency on glycolytic metabolism and might even forgo oxidative phosphorylation in the presence of oxygen. It is this often-overlooked increased glycolytic flux on the part of cancer cells that enables clinicians to effectively localize tumors and metastatic outgrowths through 2-[^18^F]fluoro-2-deoxy-D-glucose positron emission tomography (FDG PET). This phenomenon, originally described by Otto Warburg nearly a century ago, has been highlighted as one of the central tenants of tumorigenesis and an “emerging hallmark of cancer” [1]. While this switch in metabolism has been well-documented in the literature, the underlying mechanism, by which malignant cells undergo this transition, remains in question. Existing hypotheses concerning the Warburg effect posit that forgoing oxidative phosphorylation and generating energy exclusively through glycolysis are a more efficient way to generate metabolic intermediates and nucleotides for further proliferation and to drive angiogenesis in a hypoxic, acidic microenvironment. Another theory is that increased competition for shared metabolic resources in the tumor’s vicinity causes the neoplastic cells to seek the path of least resistance and opt for the “evolutionarily less efficient pathway” to generate energy. Additionally, this shift in metabolism has been shown to increase cell proliferation as long as the supply of glucose is unlimited. A recent study suggests that cells shut off mitochondrial cellular respiration by increasing the expression of mitochondrial uncoupling protein 2 (UCP2) in an effort to mitigate the effect of cytotoxic reactive oxygen species (ROS), thereby inducing the Warburg effect through UCP2 upregulation [2]. 

Genipin, a specific UCP2 inhibitor, is extracted from the *Gardenia jasminoides* Ellis, a popular shrub in the Rubiaceae family. Like *Feijoa sellowiana* fruit (pineapple guava) and *Mentha arvensis* (wild mint), which have been widely used as folk remedies and whose mechanism of pharmacological action has been elucidated through recent studies, the desiccative ripe fruits of this plant (called Zhizi in China and Chija in Korea) are well-known and frequently used not only as an excellent natural colorant, but also as an important traditional medicine for antipyretic action, relaxing blood vessels and improving blood flow [3,4,5]. Accordingly, *G. jasminoides* is used not only as an important traditional medicine, but is also applied in many other fields such as the food, textile, and chemical industries in Asian countries [5]. Genipin is formed by geniposide hydrolysed with β-glucosidases and crosslinked proteins, collagen, gelatin, and chitosan. As a result, it is able to fix biological tissues, resulting in the production of biomaterials such as artificial bones, wound dressing materials, and blood substitutes [6]. Therefore, genipin has been widely used for drug delivery due to its excellent biocompatibility, admirable biodegradability, and stable cross-linking attributes [7]. Genipin has also been used as a fingerprint collection reagent and a cross-linker for the preparation of immobilized enzymes [8,9]. In this review, the author comprehensively discusses the anticancer effects of genipin and its diverse molecular targets and also highlights its role in the modulation of cancer cell metabolism.

## 2. Uncoupling Protein 2 (UCP2) and Cancer

**Figure 1:**
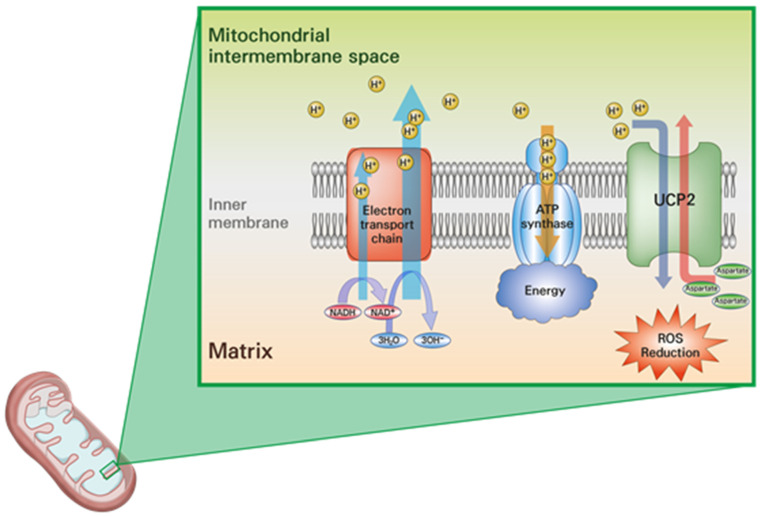
Schematic diagram of the function of uncoupling protein 2 (UCP2) as an uncoupler and an aspartate transporter in the mitochondria of cancer cells (Abbreviations: ATP: adenosine triphosphate; ROS: reactive oxygen species; NAD: nicotinamide adenine dinucleotide).

UCP2 is located on the inner mitochondrial membrane (IMM) and acts to uncouple the proton gradient across this membrane, of which the primary function is to drive ATP synthesis through ATP synthase. Under normal circumstances, protons accumulate in the inner membrane space, and ATP synthase facilitates their flow into the matrix, generating ATP in the process [10,11,12]. When UCP2 is upregulated, the opposite occurs, in that the exit of anions and protons from the matrix is facilitated. Although the mechanism by which UCP2 facilitates this proton transport is not fully understood, by allowing protons to leak across the IMM, the driving force behind ATP production through the electron transport chain is decreased, resulting in heat-energy release [2,11,13]. In addition to mitochondrial uncoupling, recent studies revealed that UCP2 plays an important role in the mitochondrial transport of C4 metabolites for NADPH production required for cancer cell growth (Figure 1) [14,15,16]. Some cancer cells, such as pancreatic ductal adenocarcinoma, have discrete pathways for growing, in which glutamine-derived aspartate is transferred into the cytoplasm, where it can be transformed into oxaloacetate by aspartate transaminase. This oxaloacetate is changed into malate and then pyruvate, enlarging NADPH/NADP+ ratio and maintaining the cellular redox state [17]. In recent years, aspartate has been progressively recognized as a serious player in the metabolism of cancer cells that utilize this metabolite for nucleotide and protein synthesis, and for redox homeostasis. Most intracellular aspartate originate from the mitochondrial catabolism of glutamate [18].

As seen in many cancers, this metabolic shift away from mitochondrial cellular respiration causes cells to increasingly depend on glycolysis to meet their metabolic demands. Consistent with its potential homeostatic role in metabolic function, UCP2 overexpression has been linked to both α- and β-cell dysfunction, and increased mRNA transcripts for UCP2 have been detected in the pancreatic islets of several animal models with type 2 diabetes [19,20,21]. A common UCP2 promoter polymorphism-866G/A has been shown to increase transcriptional activity by allowing for easier binding of the pancreatic transcription factor PAX6, increasing the risk of glucose dysregulation and type II diabetes in several human populations [22,23,24,25,26]. 

Additionally, recent studies have suggested that UCP2 has a role in global homeostatic glucose regulation due to its expression in the arcuate nucleus and proopiomelanocortin (POMC) neurons that project into the hypothalamus [27,28,29]. In addition to shifting the metabolism away from oxidative phosphorylation, UCP2 has also been implicated in increased mitochondrial Ca^2+^ sequestration, apoptotic evasion, dampened immune response, and chemotherapeutic resistance. A query of the CGGA RNA-seq and the TCGA GBMLGG database demonstrated that UCP2 expression increases with increased WHO tumor-grade and is associated with a much poorer prognosis across a cohort of brain tumors [2]. Li et al. showed that mitochondrial uncoupling affects mouse skin carcinogenesis using UCP2 homozygous knockout and wild-type mice. The knockout of UCP2 significantly suppressed the development of both benign (papilloma) and malignant (squamous cell carcinoma) tumors. UCP2 knockout mice did not exhibit increases in apoptosis during skin carcinogenesis. The oxygen consumption rates were lessened only in the carcinogen-treated UCP2 knockout mice, whereas glycolysis was augmented only in the carcinogen-treated wild-type mice. The levels of metabolites pyruvate, malate, and succinate exhibited different tendencies after carcinogen treatments between the wild-type and UCP2 knockout mice [30].

## 3. Effects of Genipin on Various Cancers

Although purine nucleotides are known to inhibit UCP2 expression under normal physiological conditions, they are not cell permeable and are unable to inhibit UCP2 when added to intact cells [31]. Zhang et al. discovered that genipin inhibits UCP2-mediated proton leaks and reverses high-glucose-induced β cell dysfunction in isolated pancreatic islets [32]. After the study was published, genipin has been used as a tool for studying UCP2 biology with numerous studies using genipin to inhibit UCP2 in cancer cells. According to Kreiter et al., genipin binds to the arginine residues of the UCP funnel, causing a decrease in the proton transporting function in the presence of long chain fatty acids. [33].The inhibition and knock out of UCP2 is a promising strategy to induce differentiation and halt cell-division or conversely facilitate apoptosis through ROS accumulation [2]. 

### 3.1. Effects of Genipin on Breast Cancer

Genipin inhibits the growing of cisplatin and tamoxifen-sensitive MCF-7 and T47D breast cancer cells by inhibiting UCP2 and inducing apoptosis and autophagy. The authors stated that UCP2 could be a therapeutic target in breast cancer patients treated with tamoxifen [34]. In another study, genipin modulated glucose metabolism and mitochondrial function in T47D breast cancer cells [35]. In this study, FDG uptake, glycolytic flux and mitochondrial oxidative respiration were repressed by genipin. [35]. In highly invasive triple-negative breast cancer MDA MB-231 cells, genipin induced apoptosis and repressed invasion and migration [36].

UCP2 over-expression also stimulates the growth of tumors in orthotopic xenograft models. MCF7-parental or MCF7-UCP2 over-expressing cells were inoculated orthotopically into the mammary fat pad of female nude mice two days after subcutaneous implantation of 17β-estradiol pellets. Tumor size was significantly greater in MCF-7-UCP2 over-expressing cells compared to MCF-7 parental cells [37].

In a recent study, a HER2-positive BT474 cell line showed that trastuzumab treatment induced the phosphorylation of HER2 and the overexpression of UCP2, and that the latter could be reversed by HER2 selective kinase inhibitor ONT-380. Moreover, UCP2 inhibitor genipin significantly enhanced the proliferation suppression effects of trastuzumab and markedly promoted apoptosis [38].

### 3.2. Effects of Genipin on Gastrointestinal Cancers

In AGS human gastric cancer cells, genipin repressed cell growth and induced caspase-3-dependent apoptosis [39,40]. Genipin also reduced the p53-independent early growth response-1 (Egr1)/p21 signaling pathway in a concentration-dependent manner [39]. In a study by Kim et al., it was determined that genipin at low doses brought nuclear factor-erythroid-2-related factor 2 (Nrf2), upregulated glutathione peroxidase, and reduced the ROS levels. However, at high doses, genipin induced cytotoxicity in the AGS cell line [41].

In Epstein–Barr virus (EBV)-associated gastric cancer SNU719 cells, genipin showed significant cytotoxicity, induced methylation on EBV C promoter and tumor suppressor gene BCL7A, arrested cell-cycle progress (S phases), upregulated EBV latent/lytic genes, stimulated EBV progeny production, activated EBV F promoter for EBV lytic activation, and suppressed EBV infection [42]. 

Genipin either alone or in combination with cisplatin inhibited HCT-116 colon cancer growth by suppressing UCP2-mediated proton leaks, promoted reactive oxygen species (ROS) formation, and sensitized cells to cisplatin [43].

Ye et al. presented that genipin significantly repressed the feasibility of HCT116 and SW480 cells in a dose- and time-dependent manner. Additionally, genipin significantly inhibited tumor growth in vivo animal models with xenografts of HCT116 and SW480 cells. The inhibition of tumor growth by genipin was combined with G0/G1 cell cycle arrest, apoptosis induction, increased ROS damage, and the loss of mitochondrial membrane potential (MMP). The expression of p53, Bax, and cleaved caspase-3 in genipin-treated cells was increased, while B-cell lymphoma-2 expression appeared to be lower in genipin-treated cells. These findings specify that genipin was capable of reducing proliferation and promoting apoptosis in colon cancer cells by inducing the p53/Bax-mediated signaling pathway [44]. Lee et al. suggested that genipin suppressed the accumulation of HIF-1α under hypoxia in HCT116 through the modulation of protein degradation, expression of VEGF, and invasion of colon cancer cells by blocking the extracellular signal-regulated kinase signaling pathway [45].

Kim et al. demonstrated that genipin inhibits the proliferation of colorectal cancer cells, and that genipin suppressed the Hedgehog pathway and increased p53 and NOXA protein levels during inhibition of Hedgehog pathway-mediated apoptosis. They also presented that p53 modulated the expression of NOXA during genipin-induced apoptosis with suppression through SMO also playing a role in this process. Subsequently, GLI1 was ubiquitinated by E3 ligase PCAF. In an xenograft tumor model, genipin suppressed tumor growth, which was also related to Hedgehog inactivation [46]. 

In a study by Hu et al., genipin crosslinked caseino-phospho-peptide (CPP)-chitosan nanoparticles (less than 300 nm) were presented to be more stable and had regulated release into the gastrointestinal tract. In this study, epigallocatechin-3-gallate was loaded on genipin cross-linked nanoparticles and demonstrated potent anti-cancer effect in in vitro models [47].

Jo et al. presented that genipin induced apoptosis and reduced Mcl-1 mRNA and protein levels in gastric cancer cell lines. Furthermore, the authors found that phosphorylation of the signal transducer and activator of transcription-3 (STAT3) is regulated by genipin. STAT3 was originally identified as a critical mediator of the IL-6-type cytokine signal pathway and described as an acute phase response factor (APRF) that can operate as a transcription factor of various cytokines, interferons, hormones, and growth factors. Additionally, the protein level of phosphor-Janus kinase 2 (JAK2) was diminished by genipin treatment, indicating that the STAT3/JAK2/Mcl-1 pathway is suppressed by genipin treatment in gastric cancer cells. Mcl-1 is closely related to mitochondrial function. These findings suggest that genipin results in a marked decrease in mitochondrial functions like MMP [48].

Kim et al. suggested that genipin sensitized cells to oxaliplatin-induced apoptosis and induced p53 expression in gastric cancer cell lines. In addition, genipin induced autophagy and LC3 expression. Knockdown of LC3 decreased cell death through the enhancement of the combination of oxaliplatin and genipin [49]. They also suggested that a combination of genipin and oxaliplatin exerts synergistic antitumor effects in vitro and in vivo in colorectal cancer cell lines through the ROS/ER stress/BIM pathway. The combination did not affect normal colon cells. BIM knockdown markedly inhibited apoptosis induced by the combination. In addition, genipin induced ROS by inhibiting superoxide dismutase 3 activity [50]. 

### 3.3. Effects of Genipin on Hepatic Cancers

In Hep3B hepatocellular carcinoma (HCC) cells, genipin induced apoptosis through NADPH oxidase-ROS-cJUN NH2-terminal kinase (JNK)-dependent activation of the mitochondrial apoptotic pathway [51]. In HepG2 and MHCC97L metastatic HCC cells, genipin treatment constrained the cellular growth and proliferation, invasion, and migration by the inhibition of MMP-2 [52]. Additionally, the anti-metastatic properties of genipin have also been investigated in in vivo models. Mice with orthotopic implantation of HCC demonstrated significant tumor size reduction without any significant change in body weight. The dissected hepatic tissue showed obvious tumor cell invasion into blood vessels in the control mice, whereas no potent intrahepatic vascular invasion was observed in the genipin-treated mice [52]. In another orthotopic HCC-implantation mouse model, oral administration of genipin significantly diminished tumor size without any obvious toxic pathologic change of lung, kidney and gastrointestinal tracts [53]. 

In addition, the numbers of CD31- and Ki67-positive cells within HCC were decreased significantly, implying inhibitory effects of genipin on angiogenesis and cellular proliferation of cancers. Furthermore, penetration of tumor-associated macrophages (TAMs) was repressed by genipin usage, which was due to its role in inactivation of inositol-requiring 1alpha (IRE1α) proteins existing on TAMs. As IRE1α brings activation of nuclear factor kappa B (NF-κB) signaling and xbox binding protein (XBP) silencing, the administration of genipin reduces inflammation in the HCC microenvironment [53].

In HCC cells, genipin suppresses STAT3 phosphorylation and nuclear translocation, which might be attributed to the binding capacity of this compound to the Src homology-2 (SH2) domain of STAT3. In addition, the therapeutic effects of genipin in a patient-derived HCC xenograft nude mice model were demonstrated [54].

Tian et al. demonstrated that the antidepressant fluoxetine increased the migratory distance of HepG2 cells by a factor of nearly 1.7 compared to the control and upregulated matrix metalloproteinases (MMPs) genes; augmented the expression of MMPs, nuclear factor kappa-light-chain-enhancer of activated B cells (NF-kB), activator protein 1 (AP-1), urokinase-type plasminogen activator (uPA), phosphorylated mitogen-activated protein kinase(P-p38), and phosphorylated protein kinase B (P-Akt); downregulated tissue inhibitor metalloproteinase (TIMP) genes; and decreased TIMP protein expression. They suggested that genipin fully counteracted the action of fluoxetine and thus could reduce the side effects [55].

Turnbull et al. suggested that palmitic acid increased superoxide production in HepG2 cells, which was amplified by genipin. Likewise, the transgenic overexpression of UCP2 in HepG2 cells prevented oxidative modifications of membranes and proteins, indicative of attenuated reactive oxygen species production [56].

Yu et al. showed that in UCP2 knockdown cholangiocarcinoma cells, the cellular proliferation and migration were suppressed, glycolysis was inhibited, and mesenchymal markers such as vimentin, snail, and Y-box binding protein-1 (YB-1) were downregulated, while AMPK was activated. The increased mitochondrial ROS and AMP/ATP ratio might be responsible for this activation. When genipin was utilized, migration and the growth of tumor cells were repressed through the enhancement of the mesenchymal-epithelial transition. Additionally, cholangiocarcinoma cells became sensitive to cisplatin and gemcitabine treatments when genipin was applied [57].

### 3.4. Effects of Genipin on Pancreatic Cancer

Interestingly, Pozza et al. revealed that UCP2 inhibition by either genipin or UCP2-specific siRNA synergistically reversed gemcitabine resistance, enhanced the activity of gemcitabine, and induced apoptosis of pancreatic cancer cells [58]. In this study, the role of UCP2 was linked for the first time to the development of chemotherapy resistance to gemcitabine. Therefore, UCP2 can be a potential biomarker for cancer resistance [58]. In pancreatic adenocarcinoma cells, genipin or UCP2 siRNA repressed cell proliferation, induced nuclear translocation of the glycolytic enzyme glyceraldehyde-3-phosphate dehydrogenase (GAPDH), and increased the expression of the autophagic marker LC3-II and autophagosomes. In addition, genipin potentiated the cytotoxic activity of gemcitabine and induced autophagic cell death [59].

In another study using pancreatic cell lines, 19 proteins were recognized as differentially expressed in Panc1 and PaCa44 cells by 2D gel electrophoresis after genipin treatment. Interestingly, UCP2 sensitized pancreatic cancer cells to the glycolytic inhibitor 2-deoxy-D-glucose, and cell growth inhibition by genipin was reversed in cells transfected with siUCP2 [60].

### 3.5. Effects of Genipin on Hematologic Malignancies

Genipin was reported to abrogate the STAT3 pathway in multiple myeloma and lymphoma cells through the upregulation of Src homology2 domain-containing phosphatase 1 (SHP-1), the endogenous inhibitor of STAT3 [61,62,63,64]. Genipin was also identified to downregulate STAT3-regulated gene products like Bcl-2, Bcl-xL, surviving, cyclin D1, and vascular endothelial growth factor, and induce apoptosis in U266, U937, and MM1S cells. Furthermore, genipin increased the cytotoxic effect of the standard chemotherapeutic agents, such as bortezomib, thalidomide, and paclitaxel in U266 myeloma cells [61]. 

Genipin inhibited cellular proliferation of K562 leukemia cells by the activation of caspase-3 and G2/M phase cell cycle arrest [65]. In another report, genipin sensitized HL-60/MX2 multidrug-resistant leukemia cells to chemotherapeutic agents such as doxorubicin and epirubicin [66].

In a recent study, novel bacterial magnetic nanoparticles holding genipin, cytosine arabinoside (Ara-C), and poly-l-glutamic acid (PLGA) inhibited the growth of HL-60 leukemia cells and brought rapid cell death [67]. In another recent study by Long et al., genipin was mixed with poly-l-glutamic acid (PLGA) as dual crosslinkers, to which cytosine arabinoside and daunorubicin were appended to produce genipin-PLGA-modified bacterial magnetosomes. When the drug-loaded magnetosomes were administered to an in vitro model of HL-60 leukemia cells, the nanoparticles showed a potent cytotoxic effect with an inhibition rate of 96% [68].

### 3.6. Effects of Genipin on Lung Cancer

In non-small cell lung cancer H1299 cells, genipin dose-dependently induced apoptosis was related to a rise in caspase-3 and caspase-9, cytochrome c, and Bax/Bcl-2 ratio [69]. In a urethane-induced carcinogenesis animal model, the administration of 50 mg/kg genipin for eight weeks significantly decreased the incidence of pulmonary adenoma from 100% to 60% and 45% in BALB/c and C57BL/6 mice, respectively. Additionally, fewer tumors were observed in the lungs of the mice [70]. The effect of genipin could be associated to its targeting of UCP2, as demonstrated by reduced UCP staining observed in the histopathological analysis of lung tissues, supplemented by reduced complex IV (cytochrome c oxidase) staining [70]. In another study, optimized gemcitabine (Gem)-loaded gelatin nanocarriers (GNCs) crosslinked with genipin (Gem-GNCs) showed selective cytotoxicity to H460 lung cancer cells but revealed lower cytotoxicity in A549 lung cancer cells [71]. In another study using A549 cells, genipin treatment reduced glucose consumption, increased ROS production, and decreased cellular survival. Combining genipin and elesclomol, an investigational drug that exerts potent anticancer activity through an increase in ROS, and a further reduction in glucose uptake and increased cellular and mitochondrial ROS production were observed. Co-treatment with genipin and elesclomol reduced colony forming capacity and cell survival. Suppression of cell survival by treatment with elesclomol and genipin was enhanced in the presence of an exogenous ROS inducer and attenuated by an ROS scavenger. The cytotoxic effects of combined genipin and elesclomol were accompanied by reduced mitochondrial membrane potential and occurred through apoptosis as demonstrated by the Annexin V assay and increased protein cleavage of PARP and caspase-3. In an A549 xenograft mouse model, tumor growth was only modestly retarded by treatment with elesclomol or genipin alone, but was markedly suppressed by the combination of the two drugs [72]. 

### 3.7. Effects of Genipin on Head and Neck Cancers

Wei et al. reported that genipin suppressed cell growth and induced apoptosis of human oral cancer cell lines. Genipin treatment upregulated the protein levels of Beclin1 and LC3II and downregulated the protein level of P62. After co-incubation with 3-MA, a kind of autophagy inhibitor, the autophagy process was lessened compared with treatment with genipin alone. Furthermore, genipin also reduced the expression of p-PI3K, p-AKT, and p-mTOR. In vivo experimentation showed that genipin significantly reduced tumor size and weight. The positive expression rate of Ki67 protein detected by immunohistochemistry was decreased, and the number of apoptotic cells measured by TUNEL assay was increased [73].

### 3.8. Effects of Genipin on Brain Tumors

In a study using U87MG and A172 glioblastoma cells, genipin moderately reduced the metabolic activity of both cell lines in a dose- and time-dependent manner. The viable cell count and colony formation ability decreased in the treated group in a concentration-dependent manner. Genipin induced apoptosis of glioblastoma cells in annexin V/PI staining assay. After genipin treatment, the expression of UCP2 and Bcl-2 was downregulated and the expression of Bax, Bak, Bik, and Cytochrome c was upregulated. The authors suggested that genipin significantly reduced the transcriptional expression of anti-apoptotic Bcl2 family members, while inducing the transcriptional expression of pro-apoptotic Bcl2 family members [74].

Zhong et al. reported that, in prolactinoma cells, genipin upregulated the expression levels of EGR1 and p21 (a downstream mediator of EGR1), with EGR1 inhibiting the proliferation, migration, and induction of prolactinoma cell apoptosis [75].

### 3.9. Effects of Genipin on Urologic Malignancies

Genipin was shown to inhibit proliferation of androgen-independent PC-3 prostate cells by suppressing UCP2, intracellular pyruvic acid, and mitochondrial succinate dehydrogenase [76].

In another study, Hong et al. stated that genipin roused mixed lineage kinase 3 (MLK3) expression in PC-3 prostate cancer cells. Furthermore, genipin-induced apoptosis was facilitated by ROS-dependent MLK3 activation [77].

In T24 and 5637 human bladder cancer cells, genipin-treated cells exhibited cell cycle arrest at the G0/G1-phase, a significant increase in the proportion of apoptotic cells, loss of MMP and Bax translocation to the mitochondria, and release of cytochrome c to the cytosol. Additionally, genipin treatment significantly reduced the phosphorylation of PI3K and Akt, significantly repressed the viability and clonogenic growing of T24 and 5637 bladder cancer cells, and inhibited the growth of T24 xenograft tumors [78].

### 3.10. Effects of Genipin on Gynecologic Malignancies

In human uterine cervix cancer HeLa cells, genipin inhibited cell proliferation and induced apoptosis dose-dependently. Apoptosis was confirmed by increase in DNA fragmentation, sub-G1 cell population, and p53 and Bax protein levels after treatment with genipin [79].

UCP2 expression in human cervical cancer cells and human ovarian serous carcinoma cells was inhibited by genipin, which it significantly augmented cisplatin sensitivity when UCP2 was repressed in vitro [80,81].

De Clercq et al. reported on the anticancer efficacy of paclitaxel-loaded genipin-crosslinked gelatin microspheres (PTX-GP-MS) for the treatment of microscopic peritoneal carcinomatosis and prevention of recurrent disease. The anticancer efficacies of IP PTX-GP-MS (PTXEtOH-GP-MS, D = 7.5 mg PTX/kg; PTXnano-GP-MS D = 7.5 and 35 mg PTX/kg), IP nanoparticular albumin-bound PTX (D = 35 mg PTX/kg), and controls (0.9% NaCl, blank GP-MS) were assessed in a xenograft mouse model with microscopic peritoneal carcinomatosis. PTXnano-GP-MS exhibited superior anticancer efficacy with significantly increased survival time and a decreased peritoneal carcinomatosis index score and ascites incidence [82,83]. 

## 4. Conclusions

In summary, UCP2 over-expression, which appears as a mechanism to protect cancer cells from excessive ROS production, is considered a target of cancer therapy without affecting normal cells. Unlike other purine nucleotides, which are difficult to use as therapeutic agents due to their low cell membrane permeability, genipin, a natural component of a plant extract known in herbal medicine, is being used for various studies of cancer treatment both in vitro and in vivo (Table 1). Although the exact molecular mechanism of action remains unclear, genipin inhibits cancer cell growth and induces apoptosis by affecting various cell signal pathways. In addition, studies are being conducted on the hypothesis that the therapeutic effect can be increased by concurrent treatment with various proven anti-cancer drugs, and research on the diversification of administration methods is also in progress. In this review, the author performed a literature review to understand that genipin, a kind of the inhibitor of UCP, can be a promising target for cancer treatment. If the precise mechanism of genipin in cancer treatment becomes clearer through future studies, and clinical trials on patients are conducted, it is hopeful that genipin can play an important role in cancer treatment. 

## Figures and Tables

**Table 1:** Anti-tumor effects of genipin on various cancers.

Authors	Types of Cancers	Effects of Genipin
Pons et al.	Breast cancer (MCF-7, T47D)	Inhibiting UCP2 and inducing apoptosis and autophagy
Cho et al.	Breast cancer (T47D)	Decreasing glycolysis and mitochondrial oxidative respiration
Kim et al.	Breast cancer (MDA MB-231)	Inducing apoptosis and repressing invasion and migration
Hua et al.	Breast cancer (BT474)	Enhancing effects of trastuzumab and inducing apoptosis
Ko et al.	Gastric cancer (AGS)	Repressing cell growth and inducing apoptosis
Kim et al.	Gastric cancer (AGS)	Inducing cytotoxicity
Son et al.	Gastric cancer (SNU719)	Suppressing EBV infection
Wang et al.	Colon cancer (HCT116)	Promoting ROS formation and enhancing effect of cisplatin
Ye et al.	Colon cancer (HCT116, SW480)	Inhibiting growth of tumor and inducing apoptosis
Lee et al.	Colon cancer (HCT116)	Suppressing accumulation of HIF-1α and invasion of cancer cells
Kim et al.	Colon cancer (HCT116)	Inactivating Hedgehog pathway and suppressing tumor growth
Jo et al.	Gastric cancer (AGS, MKN45)	Suppressing STAT3/JAK2/Mcl-1 pathway and decreasing mitochondrial function
Kim et al.	Gastric cancer (AGS, MKN45, MKN28)	Sensitizing oxaliplatin-induced apoptosis and inducing p53 expression
Kim et al.	Colon cancer (HCT116)	Enhancing effects of oxaliplatin through ROS/ER stress/BIM pathway
Kim et al.	Hepatic cancer (Hep3B)	Inducing apoptosis through mitochondrial apoptotic pathway
Wang et al.	Hepatic cancer (HepG2, MHCC97L)	Suppressing cellular growth, proliferation, invasion and migration by inhibiting MMP-2
Wang et al.	Hepatic cancer (MHCC97L)	Reducing intrahepatic invasion of cancer cells
Tan et al.	Hepatic cancer (MHCC97L)	Reducing tumor growth through suppressing IRE1α-mediated infiltration and priming of TAMs
Hong et al.	Hepatic cancer (HepG2, MHCC97L)	Suppressing STAT3 phosphorylation and nuclear translocation
Tian et al.	Hepatic cancer (HepG2)	Reducing the side effects of fluoxetine
Turnbull et al.	Hepatic cancer (HepG2)	Enhancing effects of palmitic acid to produce superoxide
Yu et al.	Cholangiocarcinoma (HuCCT1, TFK-1)	Suppressing migration and growth of tumor cells and enhancing effects of cisplatin and gemcitabine
Pozza et al.	Pancreatic cancer (PaCa44, PaCa3)	Enhancing effects of gemcitabine through UCP2 inhibition
Dando et al.	Pancreatic cancer (PaCa44)	Triggering nuclear translocation of GAPDH and inducing autophagic cell death
Brandi et al.	Pancreatic cancer (Panc1, PaCa44)	Reducing glycolysis and cellular growth
Lee et al.	Multiple myeloma (U266)	Suppressing STAT3 pathway and enhancing effects of bortezomib, thalidomide, paclitaxel
Feng et al.	Leukemia (K562)	Inhibiting proliferation through cell cycle arrest
Mailloux et al.	Leukemia (HL-60/MX2)	Enhancing effects of doxorubicin and epirubicin
Yang et al.	Lung cancer (H1299)	Inducing apoptosis
Lee et al.	Lung cancer (A549)	Enhancing effects of elesclomol
Wei et al.	Oral cancer (SCC-9, SCC-25)	Suppressing cell growth and inducing apoptosis through PI3K/AKT/mTOR pathway
Ahani et al.	Glioblastoma (U87MG, A172)	Reducing metabolic activity and inducing apoptosis
Zhong et al.	Pituitary tumor (GH3, GT1-1)	Upregulating EGR1 and Inducing apoptosis
Yao et al.	Prostate cancer (PC-3)	Inhibiting proliferation
Hong et al.	Prostate cancer (PC-3)	Increasing MLK3 expression and inducing apoptosis
Li et al.	Bladder cancer (T24, 5637)	Inducing cell cycle arrest and apoptosis
Cao et al.	Uterine cervix cancer (HeLa)	Inhibiting proliferation and inducing apoptosis
Imai et al.	Uterine cervical cancer (CaSki)	Enhancing effects of cisplatin
Kawanish et al.	Ovarian serous carcinoma (OVSAHO)	Enhancing effects of cisplatin
